# Supplementary material for: Role of Selective Digestive Decontamination in the Prevention of Ventilator-Associated Pneumonia in COVID-19 Patients: A Pre-Post Observational Study
Source: J Clin Med. 2023 Feb 10;12(4):1432. doi: 10.3390/jcm12041432 (PMC9964963; doi:10.3390/jcm12041432)
Supplement: Supplementary file 1 [file jcm-12-01432-s001.zip › jcm-2153529-supplementary.pdf]

Supplementary Materials

Table S1. Incidence of ventilator-associated pneumonia and bloodstream infection in the different waves of the pandemic.

Period	Date	Patients Included	VAP (*n*; %)	Blood Stream Infection (*n*; %)
First wave	February–June 2020	80	27 (33.7)	12 (15.0)
Second Wave	September 2020–February 2021	104	39 (37.5)	22 (21.1)
Third Wave	March–July 2021	93	27 (29.0)	20 (21.5)
Fourth wave	October 2022–March 2022	71	20 (28.2)	17 (23.9)

VAP: ventilator associated pneumonia.

Table S2. Secondary analysis in patients matched 1:1 using the individual propensity for receiving selective digestive decontamination. Demographics, comorbidities, severity scores, and laboratory at ICU admission in all the patients included in the secondary analysis and in patients with or without selective digestive decontamination. The use of steroids during ICU stay and of tocilizumab before and during ICU stay are also reported.

	All Patients (*n* = 162)	No SDD (*n* = 81)	SDD (*n* = 81)	*p* Value
Age (years; median, IQR)	66 (57–71)	66 (55–71)	64 (58–72)	0.689
Sex, male (*n*, %)	116 (71.6)	58 (71.6)	58 (71.6)	1.000
BMI (kg/m^2; median, IQR)	30 (27–34)	30 (27–35)	31 (26–33)	0.592
Comorbidities				
Diabetes (*n*, %)	40 (24.7)	20 (24.7)	20 (24.7)	1.000
Hypertension (*n*, %)	64 (39.5)	32 (39.5)	32 (39.5)	1.000
Immunosuppression (*n*, %)	30 (18.5)	15 (18.5)	15 (18.5)	1.000
SAPSII (median, IQR)	35 (30–40)	35 (30–39)	33 (29–42)	0.718
D-dimer (ng/ml; median, IQR)	1560 (888–4299)	1510 (890–2690)	1605 (888–6970)	0.446
Platelet count (10^9/L; median, IQR)	218 (155–285)	205 (147–274)	220 (171–290)	0.198
PaO2/FiO2 (mmHg; median, IQR)	92 (73–111)	97 (79–117)	88 (71–109)	0.114
Steroids (*n*, %)	162 (100.0)	81 (100.0)	81 (100.0)	1.000
Tocilizumab (*n*, %)	138 (85.2)	68 (84.0)	70 (86.4)	0.658

SDD: selective digestive decontamination; Body Mass Index: BMI; SAPSII: Simplified Acute Physiology Score II.

Table S3. Secondary analysis in patients matched 1:1 using the individual propensity for receiving selective digestive decontamination. Ventilator-associated pneumonia and other infections during intensive care stay and hospital mortality in all the patients and in patients receiving and not receiving selective digestive decontamination.

	No SDD (*n* = 81)	SDD (*n* = 81)	*p* Value
VAP (*n*, %)	31 (38.3)	22 (27.2)	0.132
Early VAP (*n*, %)	7 (8.6)	6 (7.4)	0.772
Late VAP (*n*, %)	24 (29.6)	16 (19.8)	0.145
VAP Onset time after invasive MV (days; median, IQR)	7 (5–12)	9 (5–18)	0.541
VAP Gram-stained microorganisms			0.443
Gram-positive (*n*, %)	15 (46.9)	8 (36.4)	
Gram-negative (*n*, %)	17 (53.1)	14 (63.6)	
VAP MDR microorganisms (*n*, %)	11 (35.5)	7 (31.8)	0.781
CMV blood reactivation (*n*, %)	27 (33.3)	28 (34.6)	0.868
Probable Invasive Pulmonary Aspergillosis (*n*, %)	22 (27.2)	24 (29.6)	0.727
Invasive MV length (days; median, IQR)	11 (5–25)	10 (5–33)	0.992
Hospital Length of stay (days; median, IQR)	27 (18–45)	30 (18–47)	0.874
Hospital mortality (*n*, %)	40 (50.6)	45 (58.4)	0.327

SDD: selective digestive decontamination; VAP: ventilator associated pneumonia; MDR: multidrug-resistant; CMV: cytomegalovirus; MV: mechanical ventilation.
